# Retinal Ganglion Cell Function and Perfusion following Intraocular Pressure Reduction with Preservative-Free Latanoprost in Patients with Glaucoma and Ocular Hypertension

**DOI:** 10.3390/jcm13051226

**Published:** 2024-02-21

**Authors:** Qëndresë Daka, Maja Sustar Habjan, Andrej Meglič, Darko Perovšek, Makedonka Atanasovska Velkovska, Barbara Cvenkel

**Affiliations:** 1 Department of Pathophysiology, Medical Faculty, University of Prishtina, 10000 Prishtina, Kosovo; 2 Department of Ophthalmology, University Medical Centre Ljubljana, 1000 Ljubljana, Slovenia; sustar.majchi@gmail.com (M.S.H.);; 3 Faculty of Medicine, University of Ljubljana, 1000 Ljubljana, Slovenia

**Keywords:** retinal ganglion cell, preservative-free latanoprost, electroretinogram, optical coherence tomography, optical coherence tomography angiography

## Abstract

**(1) Background**: Given the global prevalence of glaucoma and the crucial role of intraocular pressure (IOP) reduction in the management of the disease, understanding the immediate effects on retinal structure and function is essential. **(2) Methods**: This study aimed to assess the effects of preservative-free latanoprost on morphological and functional parameters in treatment-naïve patients with ocular hypertension and open-angle glaucoma. **(3) Results**: This study showed a significant reduction in IOP by an average of 30.6% after treatment with preservative-free latanoprost. Despite the significant reduction in IOP, no statistically significant changes were observed in the electroretinogram (ERG) nor the optical coherence tomography/angiography (OCT/OCTA) parameters compared to baseline. An exploration of the correlation between IOP changes and various parameters revealed a significant association solely with the macular IPL/INL plexus vessel density (VD) measured with OCTA. **(4) Conclusions**: This finding suggests a possible association between IOP reduction and changes in the macular microcirculation and provides valuable insights into the differential effects of latanoprost. Acknowledging the study limitations, this study emphasizes the need for larger, longer-term investigations to comprehensively assess the sustained effects of preservative-free latanoprost on both IOP and retinal parameters. In addition, exploring systemic factors and conducting subgroup analyses could improve personalized approaches to glaucoma treatment.

## 1. Introduction

Glaucoma, the leading cause of irreversible blindness worldwide, comprises a group of optic neuropathies characterized by the loss of retinal ganglion cells (RGCs). It is estimated that 112 million people will be affected by this disease by 2040, with open-angle glaucoma being the most common form [1,2,3,4,5].

The exact pathophysiology of glaucoma is still unknown as several risk factors and interacting processes have been described. However, it has been shown that only a reduction in elevated intraocular pressure (IOP) can successfully delay the onset and progression of the disease by preventing the loss of RGCs [6,7,8,9,10,11,12,13].

The clinical evaluation of glaucoma requires a thorough eye examination, but an assessment of optic nerve head (ONH) and retinal nerve fiber layer (RNFL) as well as visual field (VF) remain the cornerstone of glaucoma diagnosis and the most important parameters for monitoring disease progression and treatment efficacy [5,8,14]. However, these examinations remain largely subjective.

Optical coherence tomography angiography (OCTA), which has emerged as a non-invasive technique for imaging the microcirculation in the retina and choroid, is increasingly being used in glaucoma. It has been reported to be a useful tool for the assessment of optic disc and macular vessel density (VD) in retinal and choroidal slabs—biomarkers thought to be altered by disease [15]. Correlation of OCTA with structural and functional parameters is noted, while the quantitative data from OCTA appears to be useful for monitoring disease progression. Although OCTA has acceptable test–retest variability, it may have segmentation errors in the retinal layers as fixed boundaries are assigned to the slabs, while its parameters are associated with disease, individual, and eye-specific factors [15,16,17,18].

Electroretinography (ERG), which is not routinely used in clinical practice, provides objective information of the RGC function. Although RCGs are affected in patients with glaucoma before the appearance of subjective VF defects [19,20], they are not currently part of objective testing. Studies suggest that damaged RCGs can enter a dysfunctional state prior to apoptosis and can partially recover under certain conditions [21]. Therefore, the ERG has become an important tool to evaluate the effect of different treatments for glaucoma, as both the pattern ERG (PERG) and the photopic negative response (PhNR) are sensitive markers of RGC dysfunction, which is characteristic of glaucoma. The PERG is a measure of the electrical activity of the RGC population of the central retina (more than 40% of the total RGC population), whereas the PhNR reflects generalized activity of the RGCs and their axons [21,22,23,24,25,26].

Currently, the therapeutic goal in glaucoma is to prevent visual impairment by slowing the apoptosis of RGCs by lowering IOP. The treatment of first choice according to the guidelines is the prostaglandin analogue [14], one of the first in this class being latanoprost. Preservative-free latanoprost, which has fewer adverse effects and is thought to be tolerated better, has been introduced on the market [27,28]. However, data on the short-term effects on RGC function and perfusion are lacking.

While it is known that ERG responses vary depending on the level of IOP, the data on changes in OCTA parameters are still uncertain [23,24]. Knowledge of the effects of preservative-free latanoprost on ocular blood flow, a possible mechanism responsible for the development and progression of glaucoma, and on the RGC function may help to better understand the pathophysiology of the disease and treatment strategies. To our knowledge, this relationship has not yet been investigated, although OCTA and ERG are reasonable examinations to elucidate this relationship through an evaluation. Therefore, the aim of this study was to determine the changes and assess the relationship between the changes in macular vessel density (VD) in OCTA and the function of RGCs in ERG responses of patients with ocular hypertension (OHT) and primary open-angle glaucoma (POAG) 3 months after the initiation of IOP-lowering treatment with preservative-free latanoprost.

## 2. Materials and Methods

This prospective cohort intervention study was conducted in accordance with the ethical standards of the Declaration of Helsinki. The research protocol was approved by the National Ethics Committee, University Medical Centre Ljubljana, Ljubljana, Slovenia (KME 33/11/11). All patients signed a consent form after being informed about the aim of this study. This study took place from March 2022 to December 2022. The clinical examinations were performed at the Glaucoma Unit, while the electrophysiological examinations were completed at the Laboratory of Visual Electrophysiology Diagnostics, Department of Ophthalmology, University Medical Centre Ljubljana. 

### 2.1. Population

A consecutive sample of newly diagnosed POAG and high-risk patients with OHT, as defined by the European Glaucoma Society (EGS) guidelines, were considered for inclusion. To be included in this study, the following inclusion criteria had to be met: 40 years or older; best-corrected visual acuity (BCVA) ≥ 0.8 Snellen; the spherical equivalent of <5 D; cylindrical refractive error of <2 D; clear optical media; characteristic ONH changes and/or corresponding reproducible VF defects (false-positive errors < 20%, false-negative errors < 20%); early (mean defect (MD) < 6 dB) or moderate (MD 6–12 dB) glaucoma, based on the Hodapp–Parrish–Anderson (HPA) criteria; and high untreated IOP (>21 mmHg) at baseline. The diagnosis was confirmed by two glaucoma specialists, and the MD was used as a summary measure of VF. One eye per patient was randomly selected if both eyes had the same IOP; otherwise, the eye with the higher IOP at baseline was included in the statistical analysis.

Patients with primary and secondary angle-closure glaucoma, congenital and juvenile glaucoma, and those already treated with IOP-lowering modalities were not included. In addition, patients with existing retinal, macular, or other optic nerve disorders and any type of eye surgery other than uncomplicated cataract surgery as well as patients with systemic diseases affecting retinal function were excluded.

### 2.2. Clinical Examination

Patients’ demographic and medical history were collected, while a complete eye examination for glaucoma was performed before and three months after the start of IOP-lowering treatment including Snellen’s eye chart examination for BCVA; slit lamp biomicroscopy, gonioscopy, tonometry, and fundus examination after pupil dilation with 1% tropicamide (GAT-Haag-Streit BQ900 GAT, Koeniz-Berne, Switzerland); central corneal thickness (CCT) measurement (Pachmate-DGH, Tehnology Inc., Exton, PA, USA); and standard automated perimetry (SAP) with G program, dynamic strategy for diagnosis (Octopus 900 perimeter, Haag-Streit AG, Koeniz-Berne, Switzerland). Subjects were scheduled for ERG and OCT/OCTA testing within one week.

### 2.3. Electroretinography 

The clinical ERG testing was performed following the standards and guidelines of the International Society for Clinical Electrophysiology of Vision (ISCEV) [29,30] using the Espion visual electrophysiology testing system (Diagnosys LLC, Littleton, MA, USA). The PERG was recorded first without pupil dilation and by displaying a 0.8 checkerboard pattern with 99% contrast, reversing 4 times per second, on a 21.6° × 27.8° cathode ray tube screen stimulator. Patients were positioned 70 cm from the screen stimulator with optimal refractive correction. One hundred sweeps were collected for each recording and repeated at least twice. The signals were amplified and band-pass-filtered between 0.625 and 100 Hz. The average of the two most repeatable or all three repeat recordings (from 180 to 250 sweeps) was used for further analysis. Later, the pupils were dilated with 1% tropicamide (Mydriacyl, Alcon, Geneva, Switzerland) and adapted to light for 10 min. Photopic ERGs were elicited with a Ganzfeld ColorDome flash stimulator (Diagnosys LLC, Littleton, MA, USA) using 2.5 cd s/m^2^ monochromatic red stimuli (635 nm) on a 10 cd/m^2^ blue background (470 nm). The rate of the stimuli was 1 Hz, and 30 sweeps were collected for each recording, repeated at least three times. The signals were amplified and band-pass-filtered between 0.17 and 300 Hz. The average of the two most repeatable or all three repeat recordings (from 50 to 80 sweeps) was used for further analysis. 

### 2.4. OCT/OCTA 

Images of the macular region were obtained using a 100 kHz scanning speed and 1050 nm Swept-Source (SS) OCT/OCTA device (DRI OCT Triton™; Topcon Inc., Tokyo, Japan) over pupils dilated with 1% tropicamide (Mydriacyl, Alcon). The device uses OCTARA™ image processing technology, which extracts the signal changes derived from vascular flow using multiple OCT B-scans acquired at the same position. The OCT/OCTA parameters were generated using the latest integrated software (IMAGEnet6), which reduces motion artefacts and improves detection sensitivity analysis and image storage.

The scanning protocols for OCT were as follows: 7 × 7 mm^2^ area for the macula, centered on the fovea, and 6 × 6 mm^2^ for the RNFL, centered on the ONH; while for the OCTA, only a 6 × 6 mm^2^ area was used for the macular area, centered on the fovea. Only scans with an image quality of >50 were accepted. 

The integrated software was used to derive parameters of interest from the segmentation of the slabs and export them for analysis with the manufacturer’s automatic segmentation algorithm. The algorithm used for OCTA parameters automatically segmented the superficial slab 2.6 μm below the internal limiting membrane (ILM) to 15.6 μm below the inner plexiform layer (IPL), whereas vessel density (VD) was defined as the percentage of vessel area with blood flow out of the total measured area and determined by adaptive thresholding binarization. Identification was performed using an ETDRS grid overlay.

### 2.5. Outcomes 

The following outcomes were used for comparison: IOP change (mean, absolute); PERG—the P50 amplitude (measured from the N35 trough), the N95 amplitude (measured from the P50 peak), and the N95/P50 amplitude ratio; PhNR amplitude (measured from the baseline to the negative trough that clearly appeared after the b-wave and the i-wave); the PhNR amplitude ratio (PhNR/b-wave); OCT-average RNFL thickness from the peripapillary ONH (pRNFL) and macular (mRNFL) scans, and the ganglion cell layer (GCL)–inner plexiform layer (IPL) (GCL+) and RNFL+GCL+(GCL++) from the macular scans; OCTA-average macular VD for the RNFL/GCL plexus, the superficial vascular plexus (GCL/IPL plexus), and the IPL/INL plexus.

### 2.6. Statistical Analysis

All statistical analyses were performed with IBM SPSS Statistics Version 28.0 (IBM Corporation, Armonk, NY, USA). Quantitative data are described as mean (standard deviation) and percentage in continuous and numerical data. Normality of distributions for dependent variables was tested using the Shapiro–Wilk test. The two-tailed paired *t*-test was used to compare the changes in IOP, OCT, OCTA, and ERG measurements from baseline to month 3 post-therapy for all patients, with a *p* value < 0.05 considered statistically significant.

Pearson’s correlation was used to determine the relationship between changes in IOP and changes in OCT/OCTA and ERG measurements 3 months after starting treatment with preservative-free latanoprost. 

## 3. Results

Eleven eyes of eleven participants were included in this study. After baseline examinations, seven eyes were categorized as OHT, one as suspected glaucoma, one as mild POAG, and two as moderate POAG according to the HPA criteria. Six of the eleven patients (55%) were male. Mean age was 64.7 years (SD 11.2; range, 51–82 years), central corneal thickness was 580 μm (SD 30.2; range, 536–628 μm), mean baseline IOP was 24.6 mmHg (SD 3.8; range, 21–34 mmHg) with an average mean defect (MD) on Octopus perimetry of 2.8 dB (SD 3.2; range, 0–9.4 dB), and the square root of loss variance (sLV) of 4.0 dB (SD 2.2; range, 1.5–8.1 dB). The characteristics of the participants and the OCT/OCTA and ERG measurements at baseline are shown in Table 1. 

The changes in IOP, OCT/OCTA, and ERG measurements 3 months after starting IOP-lowering treatment with preservative-free latanoprost are summarized in Table 2 and Table 3. Three months after starting treatment, there was a statistically significant mean reduction in IOP of 30.6% (mean 7.5 mmHg; range 5.3–9.6 mmHg; *p* <0.001) compared to baseline IOP (Table 3).

There were no significant changes in mean values of the OCT and OCTA parameters compared to baseline (Table 3). Similarly, there were no statistically significant changes in the mean values of the selected ERG parameters (Table 3). However, with the comparison of values of the individual subject (Table 2), some showed an improvement of either PhNR or N95 amplitudes after 3 months of treatment (as seen for cases 3, 4, and 5), while, in some subjects, there was inconsistent worsening of one ERG parameter and an improvement of another (cases 8 and 10).

**Figure 1:**
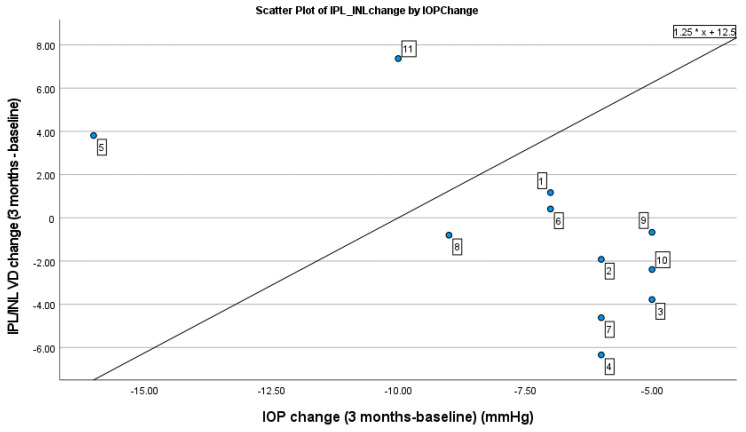
Scatterplot of VD change in OCTA IPL/INL by change in IOP from baseline values.

The correlation between the changes in IOP and the changes in OCT/OCTA and ERG measurements 3 months after the start of treatment is shown in Table 4 and Figure 1. The correlation was significant only for the VD OCTA IPL/INL plexus (*p* = 0.03). The VD in the IPL/INL plexus increased only in four patients, an only slightly in two patients, while in another two patients—cases 5 and 11—with greater IOP-lowering treatment, the increase in VD was 3.8 and 7.4%, respectively. In seven patients with an IOP reduction between 5 and 9 mmHg, there was a decrease in VD in the IPL/INL plexus (Table 4 and Figure 1).

## 4. Discussion

In our study, we investigated the response to treatment with preservative-free latanoprost in treatment-naïve patients with POAG, suspected glaucoma, and OHT, as well as the changes associated with the reduction in IOP 3 months after the start of treatment using morphological (OCT/OCTA) and functional measurements (ERG). The efficacy of prostaglandin analogues in lowering IOP has already been emphasized in the EGS guidelines. However, with our study, we aimed to contribute to the existing evidence by focusing on the effects of prostaglandin analogues on retinal parameters, an aspect that could have broader implications for glaucoma management beyond the lowering of IOP. The significant mean reduction in IOP of 30.6% was not accompanied by statistically significant changes in ERG and OCT/OCTA parameters compared to baseline. Only the change in OCTA IPL/INL correlated significantly with the change in IOP. 

Several studies have used OCTA to assess the changes in VD after lowering IOP [17,31,32,33,34,35,36,37]. Hollo [31] was the first to report a significant increase in peripapillary angioflow density at 2–4 weeks in six eyes of four young subjects after a large (more than 50%) decrease in IOP with topical medication. In a prospective study, an OCT/OCTA assessment was performed in 17 patients (33 eyes) with newly diagnosed early glaucoma before and 3 and 6 months after starting IOP-lowering medication [32]. Interestingly, in a subgroup of eyes with an IOP reduction of at least 20% (mean reduction 34%), a significant decrease in deep perifoveal VD was observed at 3 months compared to baseline. This was interpreted as a greater sensitivity to rapid IOP reduction with increased lability of the deep parafoveal tissue and caused a reduction in VD [32]. No changes were observed in other OCTA parameters. Gillmann and co-workers [33] measured the peripapillary and macular VD 2 and 6 months after selective laser trabeculoplasty in 21 patients with POAG. After 2 months, the mean IOP decreased by 15.4% (2.9 mmHg), with an increase in the parafoveal and perifoveal VD, which returned to near baseline after 6 months. These changes were independent of IOP, but the intensity of the signal strength had an effect on VD. After trabeculectomy, some studies [17,34,35,36] reported a significant increase in peripapillary and macular VD in open-angle and angle-closure glaucoma [37], which was associated with higher pre-operative IOP and greater IOP reduction [36], suggesting that ocular perfusion impairment may be improved via IOP reduction. The improvement in NFL-plexus capillary density occurred in areas with minimal RNFL thinning, in eyes with early glaucoma, which may have greater potential to restore perfusion [18], while eyes with advanced visual field loss after successful trabeculectomy had reduced peripapillary and macular VD, which may be relevant to the experience of deterioration after surgery [38,39]. Other studies found no changes in peripapillary and macular VD after a surgical reduction in IOP [40,41], while Ch’ng and co-workers [42] reported an increase in superficial foveal VD at 3 and 6 months post-operatively. A significant increase in VD was found in the deep ONH and was associated with a reduction in the curvature of the lamina cribrosa after surgically lowering IOP and not with the change in IOP [41].

In our study, two hypertensive eyes (cases 5 and 11; Figure 1) with the greatest reduction in IOP from baseline (18 and 10 mmHg, respectively) showed an increase in VD of the macular IPL/INL capillary plexus, two eyes showed a slight increase in VD, and seven eyes showed a decrease in macular VD from baseline. The reasons for the decrease in macular VD in these seven eyes despite a decrease in IOP are unclear. It would be useful to investigate the repeatability and reproducibility of our device and software in control subjects to determine whether a difference in OCTA parameter measurements in a given patient is clinically significant or within the accepted “noise” of the technology and the patient’s daily physiological variation. Studies have shown good intra-session repeatability and good inter-session reproducibility of OCTA vessel parameter measurements in healthy and glaucomatous eyes using commercial software for OCTA devices [43,44,45,46]. The inter-session coefficient of repeatability is the best measure for this assessment as it represents the estimated range in which the difference between two repeated measurements for a given eye falls 95% of the time [43]. Vorperian et al. [46], using commercial software for macular VD parameters, reported the inter-session within-eye coefficient of repeatability of 1.349 (95% CI 1.173; 1.528) for non-glaucomatous eyes and 1.767 (95% CI 1.501; 2.029) for glaucomatous eyes. This means that the absolute difference between two measurements and between two sessions should not be greater than 1.349 for non-glaucomatous and 1.767 for glaucomatous eyes in 95% of cases [46]. In addition, there may be other factors that can directly affect the retinal microcirculation, such as caffeine consumption [47], nicotine [48], or level of physical activity [49]. A short-term increase in IOP of 10–15 mmHg for 1–2 h in a suspected occludable angle after laser peripheral iridotomy and dark room prone provocative test did not affect VD in the macula or ONH as examined with OCTA [50,51]. In contrast, a sudden increase in IOP of approximately 13 mmHg (50% increase from baseline) in healthy subjects using a suction cup resulted in a significant decrease in VD in the ONH and macular (superficial and deep) capillary plexus [52]. The differences in the results may be related to the amount of IOP change, the methods of lowering IOP, the age of the patients, and the extent of glaucomatous damage. The absence of changes in OCTA findings may reflect IOP-related autoregulation within a certain range of IOP change [51,53,54], which may be the case in our study with a moderate mean IOP reduction of 30.6% from baseline. 

To evaluate the changes in function after lowering IOP, we used the PERG and the PhNR, which measure the integrity of the RGCs. Lowering IOP in eyes with OHT and early glaucoma was associated with an increase in PERG and PhNR amplitude, indicating an improvement in inner retinal function [55,56,57,58,59,60]. RGC dysfunction measured with PERG could be detected in suspected and mild glaucoma several years before the appearance of morphological changes measured with OCT [61]. Recently, PhNR was used to assess RGC function in patients with glaucoma and detected an improvement in inner retinal function after 12 weeks of oral nicotinamide supplementation compared to baseline [62]. Igawa and co-workers [63] showed an increase in PhNR amplitude in the first week after filtration surgery with a mean decrease in IOP of 50% from baseline, indicating rapid functional improvement. In our study, we found no significant changes in ERG parameters after lowering IOP. This could be due to the lowering of IOP too moderately and the higher variability of ERG measurements, which were found to be high compared to the morphological measurements with OCT, limiting the sensitivity of ERG in detecting changes. Inter-session variability/repeatability, calculated with a 95% limit of agreement, was within ±35.9% of the mean for PERG N95 amplitude and within 59.9% of the mean for PhNR [25], or even higher as reported by others [64,65]. In addition, the functional impairment of the RGCs must have been only very mild at baseline measurement, since in all but one patient (case 10) the baseline ERG amplitudes were within the normal range according to the laboratory reference range—so that a significant change after application of the treatment could not to be expected. 

The limitations of our study should be discussed. Recognizing the limitations and addressing them in future research will contribute to a more comprehensive understanding of the effects of treatment and lead to improvements in glaucoma management strategies. This study represents a case series of 11 patients with a wide age range (51–82 years), and we recognize the need for further large studies over a longer period and with a larger sample size. The inclusion of a limited number of eyes was indeed a limitation in this initial exploration, but small sample sizes are not uncommon in preliminary studies. Our primary intention was to generate hypotheses that could be relevant to future, more comprehensive research in this area. It is known that perfusion measurements correlate significantly with age [66] and can be influenced by other diseases, such as diabetes (in 2 patients) [52]. Three patients were treated for arterial systemic hypertension and their systemic medication did not change during the study, but arterial blood pressure was not measured. Park and co-workers [67] reported that in glaucomatous eyes with an optic disc hemorrhage, high systemic blood pressure was associated with a reduction in macular VD. However, none of our patients had an optic disc hemorrhage at baseline or after 3 months of treatment with latanoprost. The wide age range in our study adds to the diversity of our patient population, but we acknowledge that a larger and more varied cohort is required for a more comprehensive understanding of the potential effects of prostaglandin analogues on retinal parameters.

The findings of this study have potential clinical implications. The significant mean reduction in IOP by 30.6% suggests that preservative-free latanoprost could be an effective treatment for lowering IOP in treatment-naïve patients with POAG, suspected glaucoma, and OHT. However, the observation that there were no statistically significant changes in ERG and OCT/OCTA parameters compared to baseline despite the reduction in IOP suggests that the reduction in IOP achieved with latanoprost may not have immediately detectable effects on retinal morphology and function.

Conducting larger and longer-term studies could help to investigate the sustained effect of preservative-free latanoprost on both IOP and retinal parameters over a longer period of time. Given the potential influence of systemic factors, future research could systematically measure and analyze systemic parameters, including arterial blood pressure, to better understand their role in ocular perfusion and treatment outcomes. Subgroup analyses based on age, presence of comorbidities, or other relevant factors could help to identify potential differences in response to treatment within specific patient groups. As the association between systemic blood pressure and macular VD has been reported in glaucomatous eyes with optic disc hemorrhages, investigating this aspect in a larger cohort could provide insight into specific subgroups that may be more susceptible to such effects.

## 5. Conclusions

To summarize, we investigated the change in the morphological and functional parameters 3 months after a moderate reduction in IOP in our pilot study. Objective ERG measurements of visual function (PERG, PhNR) showed no significant changes compared to baseline. Only the OCTA IPL/INL plexus showed a significant correlation with the change in IOP.

Although this study provides valuable insights, we are aware that we must be cautious when generalizing our findings. This case series serves as a starting point for investigations, and we hope to conduct future studies that build on our preliminary findings to gain a more comprehensive understanding of the potential benefits and effects of prostaglandin analogues in relation to retinal parameters.

## Figures and Tables

**Table 1:** Clinical characteristics of participants at baseline.

No	Diagnosis	Eye	Age (Years)	Sex	BCVA Snellen	Systemic Diseases	CCT (μm)	IOP mmHg	VF MD (dB)	VF sLV (dB)
1	POAG	RE	59	F	1.0	AH GC	536	23	7	4.8
2	OHT	LE	59	M	1.0	DM AH HLD	620	25	2	5.6
3	OHT	LE	78	F	0.8	AH HLD OP	582	21	3.2	5.2
4	POAG	LE	71	F	0.8	BC HLD	589	21	4.4	2.8
5	OHT	RE	45	M	1.0	/	585	34	0.4	2.3
6	OHT	LE	66	M	1.0	/	588	22	0.6	3.4
7	OHT	LE	76	F	1.0	OP	628	24	2.1	6.6
8	SUSPECT	RE	52	M	1.0	OSP CAT	541	27	0.4	1.5
9	OHT	RE	67	F	1.0	DM HLD COPD	584	24	0	2.2
10	POAG	RE	82	M	0.9	AH BPH DD	554	23	9.4	8.1
11	OHT	RE	45	M	1.0	/	524	25	1.9	3.0

POAG = primary open-angle glaucoma; OHT = ocular hypertension; SUSPECT = primary open-angle glaucoma suspect; RE = right eye; LE = left eye; M = male; F = female; AH = arterial hypertension; GC = gastric cancer; DM = diabetes mellitus; HLD = hyperlipidemia; OP = osteoporosis; BC = breast cancer; OSP = obstructive sleep apnea; CAT = cataract; DD = depressive disorder; BPH = benign prostatic hyperplasia; COPD = chronic obstructive pulmonary disease.

**Table 2:** IOP, OCT/OCTA, and ERG measurements before (baseline) and 3 months after IOP-lowering therapy.

Case		IOP	OCT				OCTA			ERG				
		(mmHg)	pRNFL	mRNFL	GCL+	GCL++	RNFL/GCL	GCL/IPL	IPL/INL	P50	N95	N95/P50	PhNR	PhNR Ratio
1	baseline	23	65	24	53	77	22.86	25.74	34.35	4.97	5.22	1.06	17.05	0.33
	3 months	16	65	29	52	81	26.57	28.12	35.52	5.15	5.20	1.01	21.8	0.36
2	baseline	25	88	30	55	85	18.97	21.86	27.43	6.57	9.27	1.41	28.68	0.42
	3 months	19	90	30	56	86	19.16	21.65	25.51	6.24	8.17	1.31	28.19	0.42
3	baseline	21	102	44	64	108	17.45	20.96	26.55	4.84	6.38	1.32	17.29	0.33
	3 months	16	104	46	65	111	15.45	18.48	22.77	8.93	11.73	1.31	17.30	0.31
4	baseline	21	93	33	60	93	33.49	37.74	50.84	4.59	6.45	1.41	28.17	0.32
	3 months	14	93	34	60	94	29.87	34.06	44.50	4.06	5.96	1.47	24.21	0.31
5	baseline	34	112	45	73	119	26.00	30.95	36.51	7.93	9.26	1.17	24.9	0.34
	3 months	18	110	44	73	116	26.73	32.02	40.32	8.68	10.95	1.26	30.72	0.37
6	baseline	22	103	36	68	105	25.13	29.74	35.33	4.78	7.25	1.52	29.25	0.35
	3 months	15	103	37	68	105	26.61	29.83	35.74	4.63	6.97	1.51	27.98	0.36
7	baseline	24	118	44	70	114	17.10	20.66	26.61	4.79	7.64	1.59	18.26	0.24
	3 months	18	117	44	70	114	16.35	18.61	21.99	5.75	8.41	1.46	20.1	0.24
8	baseline	27	95	37	56	93	24.21	27.9	30.85	4.94	6.24	1.26	30.75	0.54
	3 months	18	100	38	58	97	24.64	28.60	30.05	5.77	8.29	1.44	17.77	0.22
9	baseline	24	108	40	63	102	15.39	18.68	23.65	5.40	8.6	1.59	25.41	0.32
	3 months	19	105	41	63	104	16.83	20.32	22.98	6.14	9.35	1.52	24.92	0.25
10	baseline	23	59	18	59	78	19.52	23.42	34.08	2.79	3.09	1.11	5.55	0.18
	3 months	15	60	20	59	79	22.43	25.84	31.69	1.54	1.68	1.09	9.62	0.33
11	baseline	25	89	38	58	96	19.50	22.55	21.52	4.02	7.01	1.74	23.95	0.51
	3 months	15	89	39	58	97	18.48	22.96	28.89	4.30	6.98	1.62	25.92	0.53

IOP = intraocular pressure; OCT = optical coherence tomography; OCTA = optical coherence tomography angiography; ERG = electroretinography; pRNFL = peripapillary retinal nerve fiber layer thickness; mRNFL = macular retinal nerve fiber layer thickness; GCL = macular ganglion cell layer; GCL+ = macular ganglion cell–inner plexiform layer thickness; GCL++ = RNFL + GCL + thickness; IPL = inner plexiform layer; INL = inner nuclear layer.

**Table 3:** Changes in IOP, OCT/OCTA, and ERG measurements 3 months after starting IOP-lowering treatment.

Parameters	Pre-Treatment (SD)	Post-Treatment (SD)	Mean Change: 3 Months–Baseline (SD) (95% CI)	*p* Value *
IOP (mmHg)	24.5 (3.6)	17.0 (1.6)	−7.5 (3.3) (−9.6 to −5.3)	<0.001
OCT				
pRNFL (μm)	97.4 (15.1)	97.6 (14.4)	0.2 (2.2) (−1.4 to 1.8)	0.78
mRNFL (μm)	37.1 (6.7)	38.2 (6.7)	1.1 (1.6) (−0.4 to 2.2)	0.06
GCL+ (μm)	62.0 (6.8)	62.3 (6.7)	0.3 (0.3) (−0.3 to 0.9)	0.23
GCL++ (μm)	99.2 (12.9)	100.5 (11.7)	1.3 (2.1) (−0.21 to 2.8)	0.08
OCTA				
RNFL/GCL (VD)	21.8 (5.2)	22.1 ((5.1)	0.3 (2.1) (−1.1 to 1.7)	0.63
GCL/IP (VD)	25.5 (5.7)	25.5 (5.4)	0.03 (2.0) (−1.3 to 1.4)	0.97
IPL/INL (VD)	31.6 (8.1)	30.9 (7.5)	−0.7 (3.9) (−2.3 to 1.9)	0.56
ERG				
P50 (μV)	5.1 (1.3)	5.6 (2.1)	0.51 (1.4) (−0.4 to 1.4)	0.25
N95 (μV)	6.9 (1.8)	7.6 (2.8)	0.7 (1.9) (−0.6 to 1.9)	0.27
N95/P50	1.4 (0.2)	1.4 (0.2)	−0.02 (0.1) (−0.1 to 0.05)	0.58
PhNR (μV)	22.7 (7.5)	22.6 (6.1)	−0.1 (5.2) (−3.5 to 3.4)	0.97
PhNR ratio	0.4 (0.1)	0.3 (0.1)	−0.02 (0.1) (−0.1 to 0.1)	0.64

* Paired *t*-test; IOP = intraocular pressure; OCT = optical coherence tomography; OCTA = optical coherence tomography angiography; ERG = electroretinography; pRNFL = peripapillary retinal nerve fiber layer thickness; mRNFL = macular retinal nerve fiber layer thickness; GCL = macular ganglion cell layer; GCL+ = macular ganglion cell–inner plexiform layer thickness; GCL++ = RNFL + GCL + thickness; IPL = inner plexiform layer; INL = inner nuclear layer.

**Table 4:** Pearson’s correlation between changes in IOP and changes in OCT/OCTA and ERG measurements 3 months after starting IOP-lowering treatment.

Change from Baseline Values		IOP Change from Baseline (mmHg)
OCT pRNFL (μm)	Pearson’s correlation	0.10
	P	0.79
OCT mRNFL(μm)	Pearson’s correlation	0.39
	P	0.26
OCT GCL+ (μm)	Pearson’s correlation	0.05
	P	0.90
OCT GCL++ (μm)	Pearson’s correlation	0.59
	P	0.07
OCTA RNFL/GCL (VD)	Pearson’s correlation	−0.03
	P	0.92
OCTA GCL/IPL (VD)	Pearson’s correlation	−0.23
	P	0.50
OCTA IPL/INL (VD)	Pearson’s correlation	−0.67
	P	0.03
PERG P50 (μV)	Pearson’s correlation	0.12
	P	0.97
PERG N95 (μV)	Pearson’s correlation	−0.12
	P	0.73
PhNR (μV)	Pearson’s correlation	−0.17
	P	0.62

## Data Availability

Data are available upon reasonable request.
